# The ASYMMETRIC LEAVES1 ortholog PagAS1a promotes xylem development and plant growth in *Populus*

**DOI:** 10.48130/forres-0025-0011

**Published:** 2025-05-23

**Authors:** Yingzi Li, Yunlong Li, Bo Zhang, Aimin Wu, Lijun Liu

**Affiliations:** 1 State Forestry and Grassland Administration Key Laboratory of Silviculture in downstream areas of the Yellow River, College of Forestry, Shandong Agricultural University, Tai'an 271018, Shandong, China; 2 Guangdong Key Laboratory for Innovative Development and Utilization of Forest Plant Germplasm, College of Forestry and Landscape Architecture, South China Agricultural University, Guangzhou 510642, Guangdong, China

**Keywords:** *Populus*, Xylem development, Plant growth, PagAS1a, MYB domain transcription factor

## Abstract

ASYMMETRIC LEAVES1 (AS1) is a central regulator of leaf polarity establishment in *Arabidopsis*. However, the functions of its ortholog genes in *Populus* are unclear. In this study, we performed gene expression analysis and found that the *AS1* ortholog gene *PagAS1a* exhibited significantly higher expression levels in the secondary phloem than in the secondary xylem within the wood formation zone. Subcellular localization analysis showed that *PagAS1a* localizes in the nuclei. Compared to the wild type, both *PagAS1a* overexpression (*PagAS1a*-OE), and dominant repression (*PagAS1a*-SRDX) plants exhibited enhanced height and stem diameter growth. Analysis of stem cross-sections revealed that both overexpression and dominant repression of *PagAS1* promoted secondary xylem development. We performed transcriptome sequencing (RNA-seq) for gene expression analysis, and identified 2,736 significantly differentially expressed genes (DEGs) in *PagAS1a*-OE plants. GO analysis revealed that genes associated with photosynthesis, lignin biosynthetic, and cellulose biosynthetic pathways were significantly enriched in up-regulated DEGs in *PagAS1a*-OE, while genes associated with lignin catabolic, and phenylpropanoid catabolic pathways were significantly enriched in down-regulated DEGs. Together, our results suggest that the *AS1* ortholog *PagAS1a* is an important regulator of secondary xylem development and plant growth in *Populus*.

## Introduction

Compared to annual herbaceous plants, extensive secondary growth is a distinctive feature of woody tree species. During secondary growth, stem cells in the vascular cambium undergo periclinal divisions, giving rise to secondary phloem on the outer side, and secondary xylem on the inner side of the stem^[1]^. Secondary xylem, which is commonly known as wood, primarily consists of fiber and vessel cells. Fibers provide strong mechanical support for the ever-increasing plant biomass, while vessels are essential for water conduction and nutrient transportation from roots to crowns^[2]^. Therefore, secondary xylem development is fundamentally important for tree growth.

Secondary cell wall (SCW) biosynthesis is a crucial step in secondary xylem development and has three major components, including 40%–50% cellulose, 20%–30% lignin, and 20%–30% hemicellulose^[3]^. Cellulose is a linear β-1,4–linked glucose polymer synthesized by the plasma membrane-localized family-2 glycosyltransferase (GT) cellulose synthase (CesA)^[4,5]^. In transmission electron microscopic visualization, the CesAs are assembled into a six-lobed rosette structure complex, termed cellulose synthase complexes (CSCs), which include 18, 24, or 36 CesAs^[6]^. Genetic and biochemical studies found there are two types of CSC, type I contains CesA4, CesA7, and CesA8, while type II contains CesA1, CesA3, and CesA6^[7−9]^. Quantitative proteomics analysis showed that the CESA stoichiometry in angiosperm tree aspen and gymnosperm tree Norway spruce is different, furthermore, the CESA stoichiometry of aspen tension wood is different from that of normal wood^[10]^. Compared to the wild type, the plant growth, SCW thickness, and cellulose content are significantly reduced in *PtrCesA4*, *PtrCesA7,* and *PtrCesA8* RNAi knockdown or Cas9/gRNA-induced knockout plants^[11]^. Lignin is a phenolic biopolymer that is hydrophobic and plays a critical role in vascular transport. Lignin is polymerized from three major monolignols: guaiacyl (G), syringyl (S), and 4-hydroxyphenyl (H) monolignol. In dicotyledon woody trees, S and G are the most abundant lignin subunits, and the S/G ratio significantly affects wood properties. The monolignol biosynthetic pathway is well-characterized, including 23 enzymes and 24 metabolites^[12−13]^. Lignin polymerization is catalyzed by laccases and peroxidase^[14,15]^. Genetic studies demonstrated that suppression or knockout of monolignol biosynthesis genes significantly reduce lignin content^[16−18]^, and down-regulation of laccases genes also lead to significantly reduced lignin content and SCW structure^[19−21]^.

The biosynthesis of cellulose and lignin is tightly controlled by a hierarchical transcriptional network^[22,23]^. Many studies have demonstrated that MYB transcription factors are key regulators directly regulating the expression of cellulose and lignin biosynthesis genes^[24−28]^. ASYMMETRIC LEAVES1 (AS1) is an R2R3 type MYB transcription factor that promotes adaxial identity in cotyledons, leaves, and floral organ development^[29,30]^. In *Arabidopsis*, AS1 forms a complex with AS2 and represses the expression of class I KNOX genes during the establishment of adaxial-abaxial polarity in leaf primordia^[30]^. The AS1–AS2 complex represses abaxial genes through both Polycomb-dependent and independent mechanisms^[31−33]^. In addition, mutation of *AS1* and *AS2* also cause abnormal midvein development^[34]^. A recent study demonstrated that overexpression of *AS1* promotes the elongation of abaxial petiole cells through activating the auxin biosynthesis and signaling pathways during leaf hyponasty growth, and *as1* mutants display reduced hypocotyl growth under shade conditions in *Arabidopsis*^[35]^. These studies suggested that *AS1* may also play an important role in regulating vascular system development.

In this study, we functionally characterized the *AS1* ortholog gene *PagAS1a* in *Populus*. Sequence analysis revealed that the PagAS1a protein has a highly conserved R2R3 type MYB domain structure. Genetic analysis showed that both overexpression and dominant repression of *PagAS1a* promote xylem development and plant growth. Transcriptome analysis revealed the lignin and cellulose biosynthesis pathways are significantly up-regulated in *PagAS1a* overexpression plants. Together, our results demonstrate that *PagAS1a* promotes xylem development in *Populus*, which may lead to increased plant growth.

## Materials and methods

### Plant materials and growth conditions

All experiments used the hybrid poplar (*Populus alba* × *Populus glandulosa*) clone 84K. The plants were grown under a 16 h light/8 h dark cycle at 25 °C. All plants were propagated asexually, and 1-month-old seedlings were transplanted into the soil for phenotypic analysis.

### Phylogenetic analysis

Phytozome (https://phytozome.jgi.doe.gov/pz/portal.html) and NCBI (www.nlm.nih.gov) were used to retrieve the protein sequences of AS1 from different species. Phylogenetic analysis was performed using MEGA 5.0 software^[36]^. Sequence alignment was conducted with ClustalW, and the phylogenetic tree was constructed using the neighbor-joining method with 1,000 bootstrap replicates.

### Subcellular localization of PagAS1a

The coding sequence (CDS) of *PagAS1a* was amplified with gene-specific primers (Supplementary Table S1) and cloned into the pROKII vector, which contains a GFP reporter gene. The recombinant plasmid *35S::PagAS1a-GFP* and the control plasmid *35S::GFP* were transformed into *Agrobacterium* (GV3101) and injected into *Nicotiana benthamiana* leaves, respectively. The nuclei were stained with DAPI and observed under an Olympus BX53 microscope.

### RNA extraction and Quantitative Reverse Transcription Polymerase Chain Reaction (RT-qPCR)

Tissue samples, including xylem, phloem, cortex, roots, leaves, and petioles were collected from 2-month-old wild-type (WT) plants. Phloem and xylem samples were collected by stripping the bark and scraping off the phloem or xylem flanks. Total RNA was extracted using the cetyltrimethylammonium bromide (CTAB) method^[37]^. RNA (1 μg) was reverse-transcribed using the HiScript II Q RT Super Mix for RT-qPCR Kit (Vazyme, R233-01). The RT-qPCR experiments were performed using ChamQ Blue Universal SYBR RT-qPCR Master Mix (Vazyme, Q312-02), and normalized gene expression levels were determined using the ΔΔCt method, employing the *Actin* gene as the internal reference. The primers used for RT-qPCR are listed in Supplementary Table S1. At least three replicates were performed.

### Gene cloning and plant transformation

The full-length CDS of *PagAS1a* was amplified from 84K plants by RT-PCR. The construct for *35S::PagAS1a* was generated by ligating the coding sequence into the pZP211-35S-PolyA vector. The construct for dominant repression of *PagAS1a* was generated by ligating the coding sequence into the pBI121-SRDX vector. All constructs were verified by sanger sequencing. Transgenic plants were generated by *Agrobacterium* (GV3101)-mediated transformation of leaf disks as previously published^[38]^.

### Cross-sectioning and histological staining

The 7^th^ and 13^th^ internodes from 2-month-old plants were collected for stem cross-sectioning. Sections were stained with 0.1% phloroglucinol solution (10 mL pure ethanol, 1.6 mL HCl, 0.01 g phloroglucinol) for 10 min. Images of the cross-sections were captured using an Olympus BX53 microscope.

### Quantification of lignin and hemicellulose

The whole 12^th^−20^th^ stem internodes of 2-month-old WT and *PagAS1a* transgenic plants were collected for quantitative analyses of lignin and hemicellulose contents. The plant materials were baked at 80 °C for 48 h. 0.01 g of dried material from each sample was used to quantify lignin content. Lignin was extracted using the Lignin Content Detection Kit (Solarbio, BC4205), and the absorbance (A) values of the sample and the blank control were determined with a microplate reader at 280 nm to calculate the lignin content using the following formula: Lignin content = 1.3105 × ΔA/W, where *A* is the absorbance value and W is the sample weight. 0.02 g of dried material from each sample was used to quantify hemicellulose. Hemicellulose was extracted using the Hemicellulose Content Kit (Geruisi, G0716W48), and the absorbance (A) values of the sample, standard, and blank control was determined with a microplate reader at 460 nm to calculate the hemicellulose content using the following formula: Hemicellulose content = 2.61 × ΔA/(A standard × A blank)/W, where *A* is the absorbance value and W is the sample weight.

### Transient expression assay

Each effector and reporter plasmid was independently transformed into *Agrobacterium* (GV3101). *Agrobacterium* transformed with the indicated effector and reporter was cultured to an optical density (OD_600_) of 0.8, mixed at a ratio of 1:1, and then infiltrated into *Nicotiana benthamiana* (tobacco) leaves. The firefly luciferase (LUC) and Renilla luciferase (REN) activities were measured after 48 h incubation.

### RNA sequencing (RNA-seq) and data analysis

The 7^th^ to 12^th^ internodes of whole stems were collected from 2-month-old WT and *PagAS1a*-OE (OE11) transgenic plants for RNA extraction. The Illumina X Ten platform was used for 150 bp paired-end RNA sequencing. The genome assembly version 3.0 of *Populus trichocarpa* was used in RNA-seq analysis. Clean sequencing reads were mapped to the genome using hisat2 with default parameters^[39]^, the mapped reads were counted using htseq-count^[40]^, and significantly differentially expressed genes (DEGs) were identified using the edgeR package^[41]^ with a Fisher's exact test FDR of less than 0.05. Each sequencing sample included three biological replicates.

### Accession number

Accession numbers for genes used in this study are as follows: PtrAS1a (Potri.004G102600), PtrAS1b (Potri.017G112300), PtrAS1c (Potri.006G085900), PagAS1a (Pag.A04G001079), PagAS1b (Pag.B17G000462), PagAS1c (Pag.A06G002169), AtAS1 (AT2G37630), OsAS1 (LOC_Os12g38400), ZmAS1 (Zm.01G232800), TaAS1a (Traes_5BS_E635B0A281), TaAS1b (Traes_5DS_8F3BD4450), TaAS1c (Traes_5BS_E635B0A281), BpAS1a (BPChr11G06937), BpAS1c (BPChr14G12721), MdAS1a (MD17G1073900), MdAS1c (MD12G1142500), SpAS1a (SapurV1A.0101s0440), SpAS1c (SapurV1A.0001s1780), TcAS1a (Thecc1EG019507t1), TcAS1c (Thecc1EG021932t1), GmAS1a (Glyma.03G081900), GmAS1c (Glyma.18G181300), SlAS1 (Solyc09g010840).

## Results

### Characterization of *PagAS1a*

**Figure 1:**
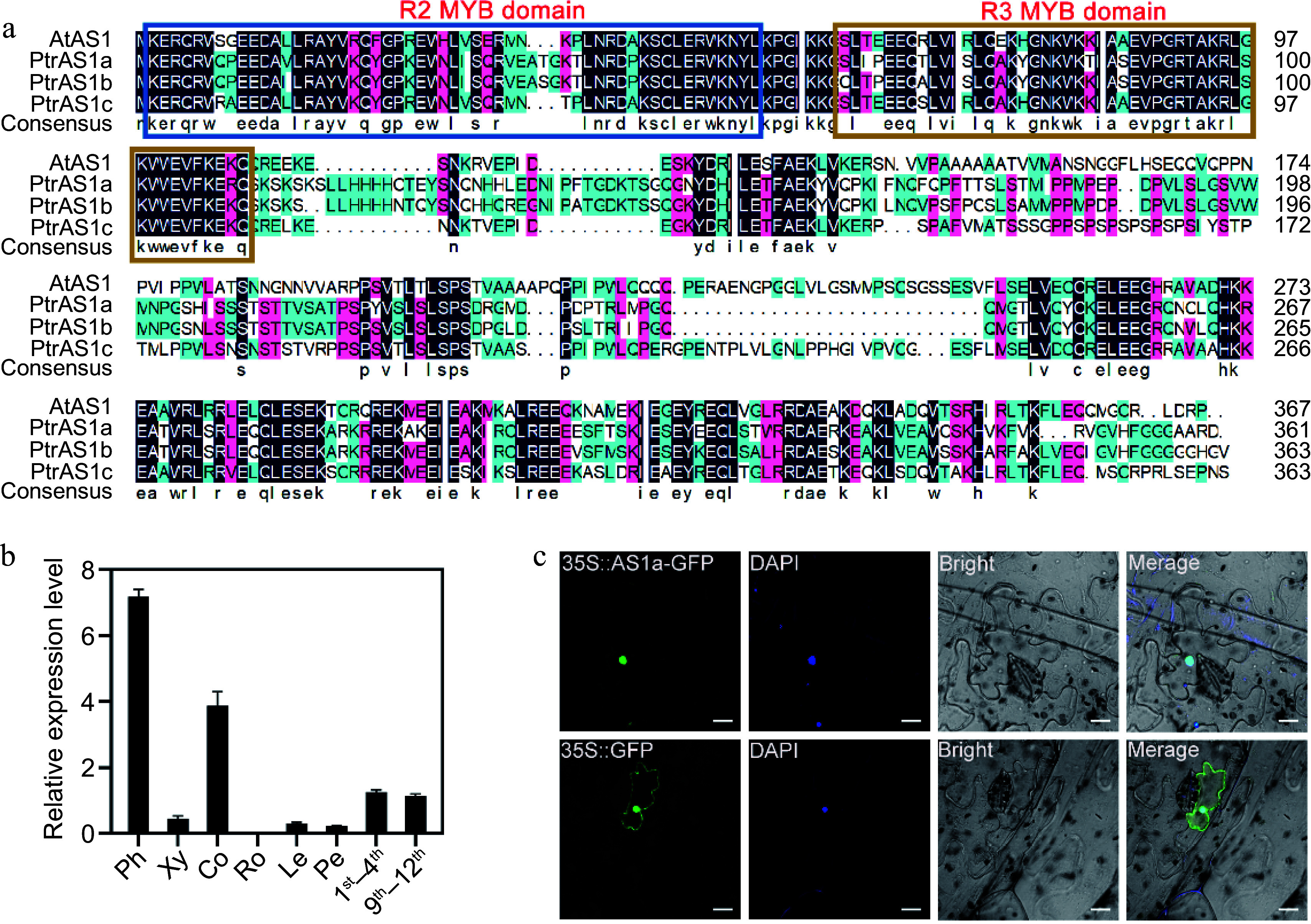
Characterization of *PagAS1a*. (a) Multiple sequence alignment of *Populus* and *Arabidopsis* AS1 proteins. (b) *PagAS1a* expression levels in various tissues assayed by RT-qPCR. Ph, phloem; Xy, xylem; Co, cortex; Ro, root; Le, leaf; Pe, petiole; 1^st^−4^th^, first to fourth stem internodes; 9^th^−12^th^, ninth to twelfth stem internodes. (c) Subcellular localization of PagAS1a. The nucleus was confirmed by DAPI staining. Scale bar: 20 μm.

MYB transcription factors have a highly conserved MYB domain, which is composed of multiple incompletely repeated amino acid sequences (R). Based on the number of R repeats, MYB transcription factors are divided into four categories: 1R-MYB, R2R3-MYB, R1R2R3-MYB, and 4R-MYB^[42]^. The main MYB transcription factors in plants are R2R3-MYB transcription factors^[43]^. In our previous RNA-seq analysis of differentially expressed genes (DEGs) between the secondary xylem and secondary phloem in *Populus*, we found that the *PagAS1a*, an ortholog gene of *AS1*, expressed significantly higher in the secondary phloem than in the secondary xylem (Supplementary Fig. S1)^[44]^. Sequence analysis showed that PagAS1a is a R2R3-MYB transcription factor which contains highly conserved R2R3 repeats in the DNA-binding domain in the N-terminal region (Fig. 1a). To further characterize the *PagAS1a* gene, we evaluated the expression of *PagAS1a* in xylem, phloem, cortex, root, leaf, petiole, 1^st^ to 4^th^ internodes, and 9^th^ to 12^th^ internodes by RT-qPCR (Fig. 1b). The results showed that *PagAS1a* was expressed to varying degrees in different tissues and the expression level was highest in the phloem, which was consistent with the RNA-seq results.

To examine the subcellular localization of PagAS1a, we constructed a vector expressing the PagAS1a-GFP fusion protein and transiently expressed it in tobacco leaves. Our results showed that the PagAS1a-GFP fusion protein was specifically distributed in the nucleus, while the GFP alone was distributed in both nucleus and cytoplasm (Fig. 1c), indicating that PagAS1a is a nuclear-localized protein.

### Phenotype of *AS1a* overexpression and dominant repression transgenic plants

**Figure 2:**
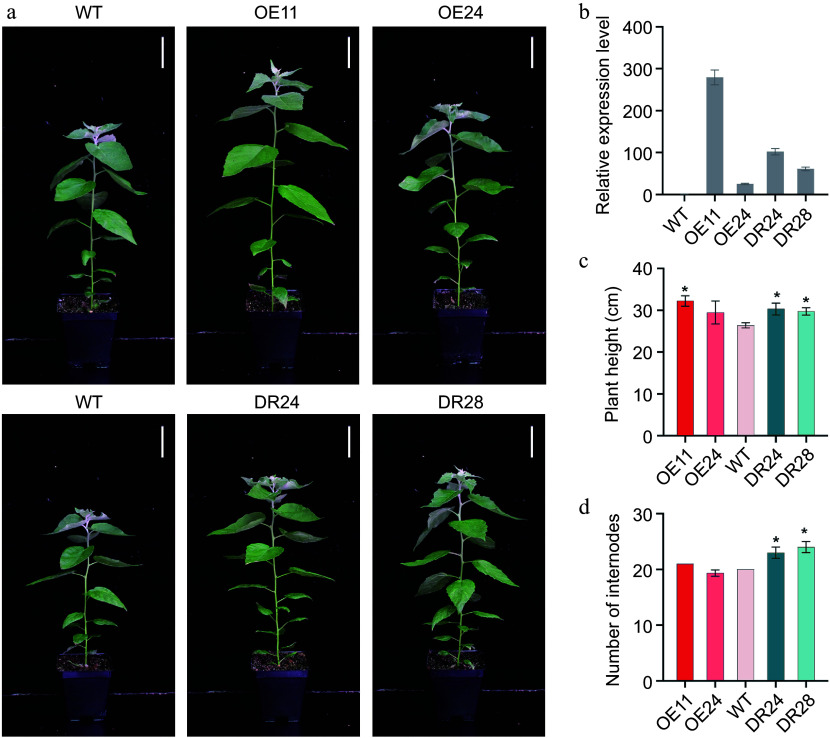
Phenotype of *PagAS1a* overexpression and dominant repression transgenic plants. (a) Morphological phenotype of 2-month-old *PagAS1a-*OE transgenic plants (OE11, OE24) and *PagAS1a-*SRDX transgenic plants (DR24, DR28) compared with WT. Scale bar: 4 cm. (b) Expression levels of *PagAS1a* in *PagAS1a-*OE and *PagAS1a-*SRDX plants determined by RT-qPCR. (c) Plant height, and (d) number of internodes of transgenic lines and wild type (WT). Error bars represent SD. Asterisks indicate significant differences between WT and transgenic lines (Student's t-test): * *p* < 0.05.

To functionally characterize the *PagAS1a* gene, we generated its overexpression transgenic plants (*PagAS1a-*OE) under the control of the cauliflower mosaic virus (CaMV) 35S promoter in *Populus*. We also employed the chimeric repressor silencing technology (CRES-T) to construct *PagAS1a* dominant repression lines (*PagAS1a-*SRDX) by fusing the *PagAS1a* coding sequence with the well-defined SRDX repressor domain (*PagAS1a-*SRDX) and transformed it into *Populus*. Two representative lines of *PagAS1a-*OE (OE11, OE24) and *PagAS1a-*SRDX (DR24, DR28) were selected for further analysis (Fig. 2a). The line OE11 exhibited the highest *PagAS1a* expression level, which was 279.6-fold increase relative to WT, while the line OE24 displayed a 25.1-fold increase relative to WT. The *PagAS1a* expression level in dominant repression lines DR24 and DR28 was elevated 102-fold and 61-fold relative to WT, respectively (Fig. 2b). Compared to WT, the *PagAS1a-*OE and *PagAS1a-*SRDX lines displayed significantly greater plant height (Fig. 2c). The differences were positively correlated with *PagAS1a* expression levels in different transgenic lines. No significant differences in leaves were observed between WT and *PagAS1a* transgenic plants (Supplementary Fig. S2). Notably, *PagAS1a*-SRDX plants exhibited a significantly greater number of internodes than both WT and *PagAS1a-*OE plants (Fig. 2d). These results suggested that *PagAS1a* overexpression and dominant repression have both similar and divergent functions in regulating plant growth.

### Both *AS1a* overexpression and dominant repression plants promote xylem development

**Figure 3:**
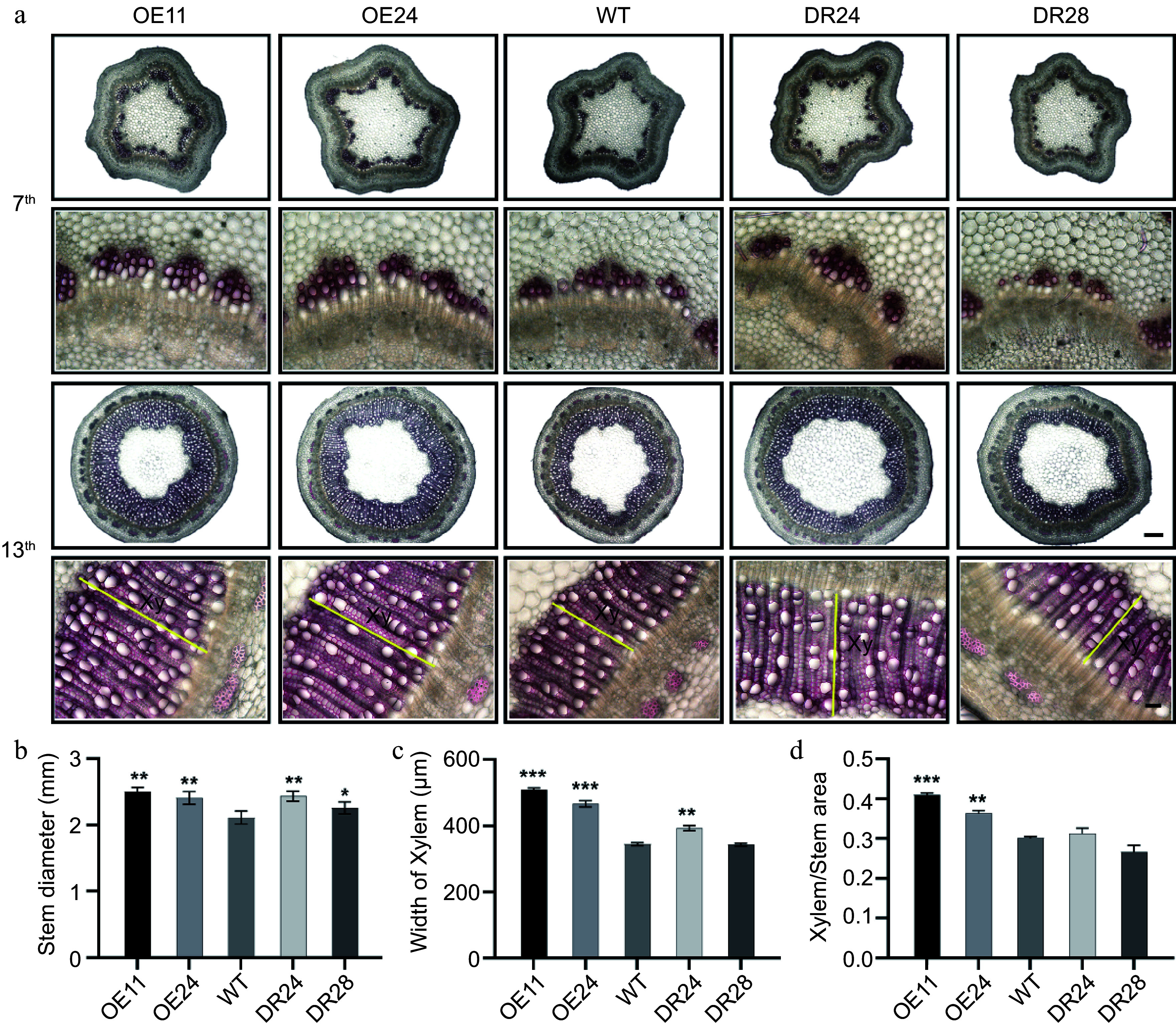
Both *PagAS1a* overexpression and dominant repression plants promote xylem development. (a) Cross-section of the 7^th^ and 13^th^ stem internodes of WT, *PagAS1a-*OE, and *PagAS1a-*SRDX plants stained with 0.1% phloroglucinol solution, yellow lines label the secondary xylem (Xy) region. Scale bars: 300 μm for upper panels and 50 μm for lower panels. (b) Stem diameter of WT, *PagAS1a*-OE (OE11, OE24), and *PagAS1a*-SRDX (DR24, DR28) plants. Quantification of (c) xylem width, and (d) xylem area/stem cross-sectional area shown in (a). Data in (b)−(d) were collected from at least three individual plants for WT and transgenic lines. Asterisks indicate significant differences between WT and transgenic lines (Student's t-test: * *p* < 0.05; ** *p* < 0.01; *** *p* < 0.001.

To further dissect the function of *PagAS1a* during secondary growth in *Populus*, we performed cross-section analyses of stems from WT, *PagAS1a-*OE (OE11, OE24), and *PagAS1a-*SRDX (DR24, DR28) plants. At the 7^th^ internode, the *PagAS1a* transgenic plants showed enhanced lignification compared to WT, with the most pronounced increase observed in OE11, the line exhibiting the highest transgene expression level (Fig. 3a). At the 13^th^ internode, the *PagAS1a* transgenic plants exhibited significantly wider stem diameter and secondary xylem compared to WT (Fig. 3a). Quantitative analyses showed that the stem diameter (Fig. 3b), xylem width (Fig. 3c), and xylem area/stem cross-sectional area (Fig. 3d) of *PagAS1a-*OE (OE11, OE24) and *PagAS1a-*SRDX (DR24) plants were significantly increased compared to WT. There were no significant differences in secondary phloem between WT and *PagAS1a* transgenic plants (Supplementary Fig. S3). We also quantified the content of lignin and hemicellulose, which are two main components of the secondary cell wall (SCW) in the secondary xylem. Consistent with the enhanced secondary xylem development, our results showed that both lignin and hemicellulose levels were significantly elevated in *PagAS1a* transgenic plants (Supplementary Fig. S4). Notably, the xylem/stem area ratio and lignin content in *PagAS1a-*SRDX line DR28, which had the low transgene expression level, were similar to WT (Fig. 3b−d). Together, these results suggested that both *PagAS1a* overexpression and dominant repression function similarly in promoting xylem development.

### PagAS1a is a transcriptional repressor

**Figure 4:**
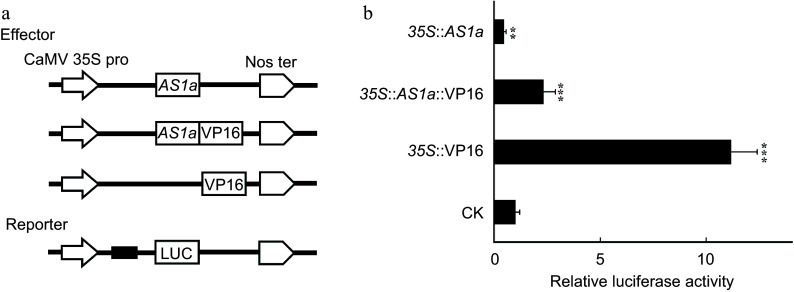
PagAS1a is a transcriptional repressor. The *PagAS1a* construct driven by the CaMV 35S promoter was used as the effector. The relative luciferase activity was measured 48 h after injection. The value of the mock group injected with the reporter and corresponding empty effector construct was set to 1. Student's t-test was performed for significance with three biological replications (** *p* < 0.01; *** *p* < 0.001).

Since the *PagAS1a* overexpression and dominant repression plants exhibited similar phenotypes in plant growth and xylem differentiation, we speculated that PagAS1a functions as a transcriptional repressor. To test this hypothesis, we performed transient expression assays by co-transfection of the reporter and various effector constructs in tobacco leaves (Fig. 4a). As shown in Fig. 4b, the relative luciferase activity in samples transfected *35S::AS1a* was significantly lower than in samples transfected the vector control (CK), suggesting that PagAS1a acts as a transcriptional repressor. To further confirm this result, we fused the *PagAS1a* with VP16 (the activation domain from Herpes simplex virus). The relative luciferase activity in samples transfected *35S::AS1a-VP16* was significantly lower than in samples transfected *35S::VP16*, supporting the conclusion that PagAS1a functions as a transcriptional repressor.

### Transcriptome analysis of *PagAS1a-*OE plants

**Figure 5:**
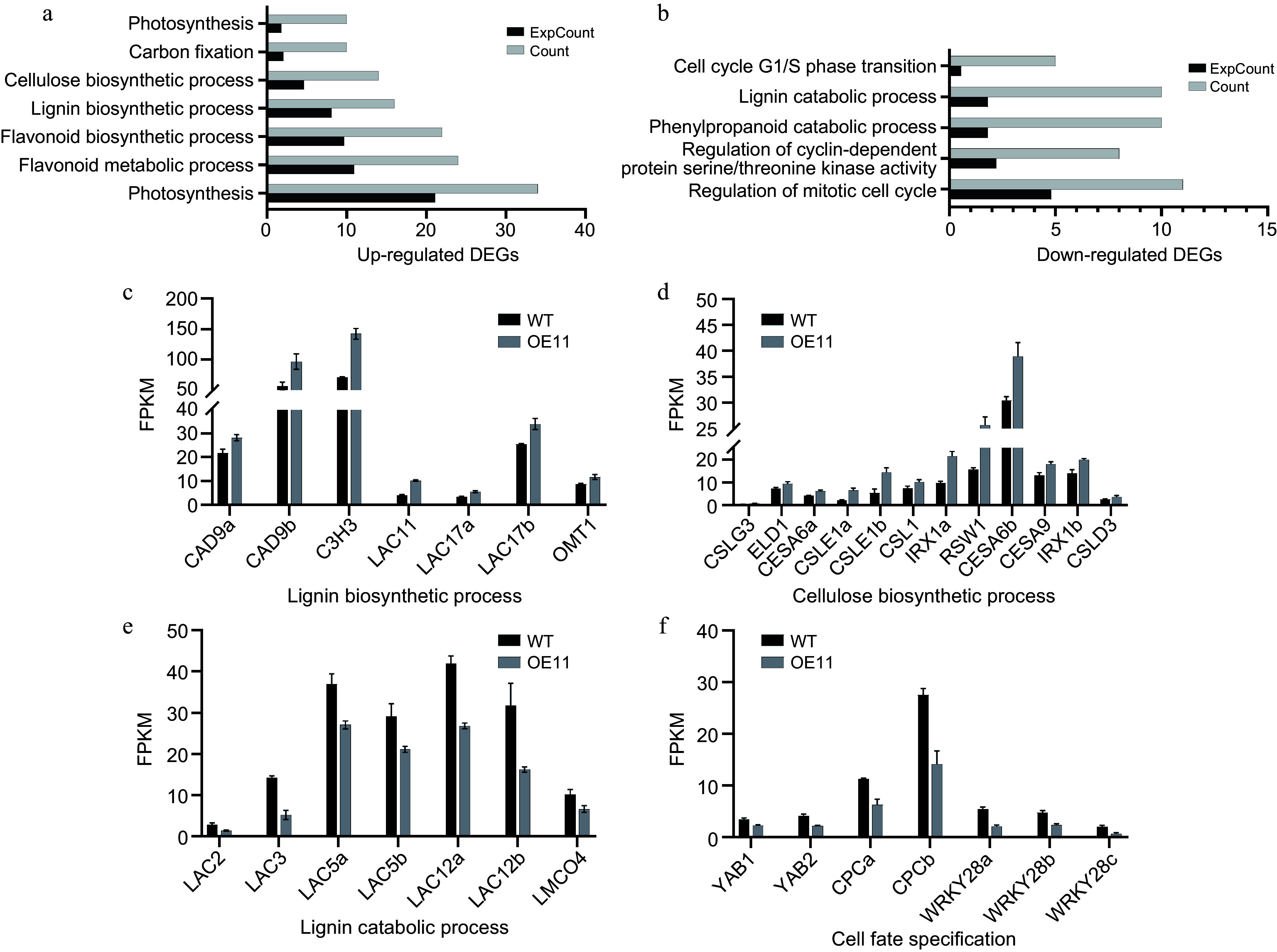
Transcriptome analysis of *PagAS1a* overexpression plants. Significantly enriched GO categories in the (a) up-regulated, or (b) down-regulated DEGs in *PagAS1a*-OE transgenic plants (OE11). Expression of DEGs involved in (c) lignin biosynthetic process, (d) cellulose biosynthetic process, (e) lignin catabolic process, and (f) cell fate specification. The average fragments per kilobase of exon per million fragments mapped (FPKM) for each gene from RNA-seq experiments are shown.

To identify genes regulated by *PagAS1a* during secondary growth, we collected whole stems from the 7^th^ to the 12^th^ internodes of *PagAS1a-*OE (OE11) and WT plants for RNA sequencing (RNA-seq). A total of 2,736 DEGs were identified between OE11 and WT (Supplementary Table S2), of which 1,669 genes were significantly up-regulated and 1,067 genes were significantly down-regulated in OE11. GO analysis revealed that 220 and 163 biological pathways (BP) were significantly enriched in the up-regulated and down-regulated DEGs, respectively (Supplementary Tables S3, S4). Consistent with the increased plant growth and advanced xylem development in *PagAS1a-*OE plants, the up-regulated DEGs in OE11 were significantly enriched in pathways including carbon fixation (GO:0015977), photosynthesis (GO:0015979, GO:0019685), lignin biosynthetic process (GO:0009809), flavonoid metabolic process (GO:0009812), cellulose biosynthetic process (GO:0030244), and flavonoid biosynthetic process (GO:0009813) (Fig. 5a; Supplementary Table S3). In contrast, the down-regulated DEGs in OE11 were significantly enriched in pathways such as the lignin catabolic process (GO:0046274), phenylpropanoid catabolic process (GO:0046271), and cell cycle-related categories (GO:0044843, GO:0007346, GO:0000079) (Fig. 5b; Supplementary Table S4). Detailed inspection showed that lignin biosynthetic genes, including *CAD9*, *C3H3*, *LAC11*, and *LAC17*^[45−48]^, were significantly up-regulated in OE11 (Fig. 5c; Supplementary Table S5). Similarly, cellulose biosynthesis genes, including *CESA6* and *IRX1*^[7,49]^, were also significantly up-regulated in OE11 (Fig. 5d; Supplementary Table S5). In contrast, genes involved in lignin catabolic pathways, including *LAC3*, *LAC5*, and *LAC12*^[21,50−54]^, were significantly enriched in down-regulated in OE11 (Fig. 5e; Supplementary Table S5). Interestingly, genes associated with cell fate specification, including *YAB1*, *YAB2*, and *CPC*^[55−58]^, were also significantly enriched in down-regulated in OE11 (Fig. 5f; Supplementary Table S5). We also inspected the expression of genes related to vascular cambium maintenance and secondary phloem/xylem differentiation. The key regulatory genes *PtaLBD1*, *PtWOX4a*, *CLE41b*, and *XND1c*, were significantly down-regulated in OE11^[59−61]^, while *PtrHB8* and *XCP1* were significantly up-regulated in OE11^[25,62,63]^ (Supplementary Fig. S5). Together, the transcriptome analysis supported the observation that overexpression of *PagAS1a* promotes plant growth and secondary xylem development.

### Phylogenetic analysis of AS1 ortholog proteins in plants

**Figure 6:**
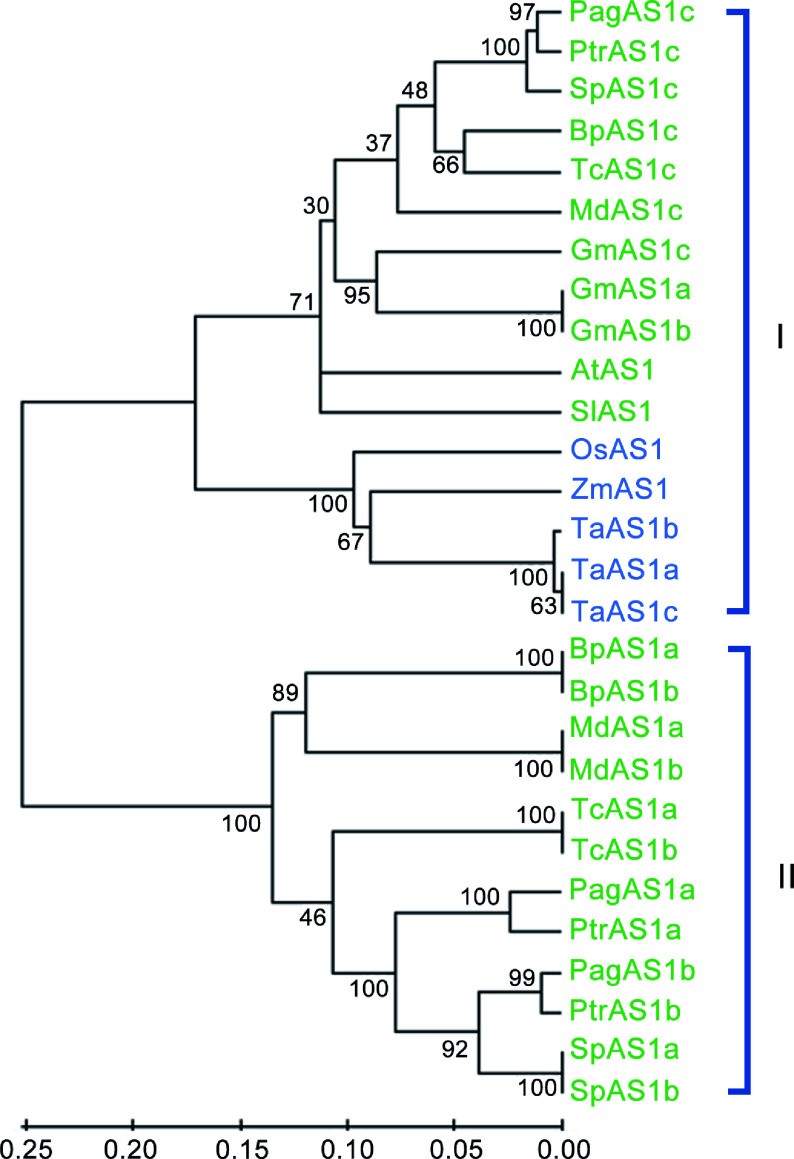
Phylogenetic analysis of *AS1* ortholog genes from 12 representative species. The neighbor-joining (NJ) tree was constructed based on the alignment of full-length protein sequences. MEGA 5.0 was used with 1,000 bootstrap replicates. Dicot species and monocot species are represented in green and blue fonts, respectively.

We identified three *AS1* orthologs genes in *Populus*: *PagAS1a* (Potri.004G102600), *PagAS1b* (Potri.017G112300), and *PagAS1c* (Potri.006G085900). To elucidate the evolutionary relationships among AS1 orthologs, we identified and isolated 28 AS1 ortholog proteins from 12 representative species, including three monocot species (*Zea mays*, *Oryza sativa,* and *Triticum aestivum*) and nine dicot species. The dicot species included three herbaceous species (*Glycine max*, *Solanum lycopersicum,* and *Arabidopsis thaliana*) and six woody species (*Betula platyphylla*, *Malus domestica*, *Populus alba × Populus glandulosa* (84K), *Populus trichocarpa*, *Salix purpurea*, and *Theobroma cacao*). We then constructed a phylogenetic tree using the full-length protein sequences with the neighbor-joining method. The results revealed that these 28 AS1 orthologs were divided into two subgroups (Fig. 6). Interestingly, subgroup I contain AS1 orthologs from all species, while subgroup II only contains AS1 orthologs from woody plants, indicating that there might be functional divergence between these two subgroups. Furthermore, a clear boundary was observed between monocot and dicot species within the subgroup I. These results indicate that the *AS1* ancestral gene existed before the divergence of monocot and dicot species.

## Discussion

Polarity establishment plays a key role in lateral organ formation. In leaves, AS1 and class III HD-ZIPs serve as essential adaxial regulators for the establishment of leaf adaxial-abaxial polarity^[32,64]^. Similarly, in stem secondary growth, the differentiation of the secondary phloem (outward) and secondary xylem (inward) from the vascular cambium represents another form of polarity establishment. Previous studies have shown that several class III HD-ZIPs transcription factors are important regulators of vascular cambium cell maintenance and differentiation during secondary growth in *Populus*^[65−68]^, suggesting that plants may employ a similar set of genes to establish polarity in both leaves and stems.

There are three *AS1* orthologs in total, *PagAS1a* (Potri.004G102600), *PagAS1b* (Potri.017G112300), and *PagAS1c* (Potri.006G085900) in *Populus*, all of which encode proteins with a conserved R2R3 MYB domain (Fig. 1a). Our previous transcriptome analysis of secondary phloem and secondary xylem showed that all these three *AS1* ortholog genes expressed significantly higher on the secondary phloem side, and the *PagAS1a* has relatively higher expression than *PagAS1*c but lower than *PagAS1b* (Supplementary Fig. S1). It should be noted that we collected samples by peeling the bark, then scraped the bark side as the secondary phloem (which included most cambium cells), and the stem side as the secondary xylem. Therefore, the secondary phloem samples in our transcriptome data were mixed with cambium samples. Further analysis with transcriptome data from AspWood showed that *PagAS1b* was highly expressed in the secondary phloem and cambium zone, while *PagAS1c* was expressed across the whole wood forming zone except the expanding xylem region (Supplementary Fig. S1)^[69]^. For unknown reasons, *PagAS1a* was not included in the AspWood dataset. In the future, carrying out RNA in-situ hybridization and native promoter-driven reporter gene analysis would be helpful to characterize the native expression of these genes more precisely.

We selected *PagAS1a* for further characterization in *Populus*. As expected, PagAS1a is localized in the nucleus (Fig. 1c). We obtained *PagAS1a* overexpression and dominant repression plants for functional analysis. As shown in Fig. 2, both *PagAS1a* overexpression and dominant repression transgenic plants displayed significantly faster growth than WT, suggesting that PagAS1a and PagAS1a-SRDX function similarly in regulating poplar growth. These results suggest that PagAS1 may function as a transcriptional repressor as PagAS1a-SRDX. We also performed cross-sectional analysis to determine whether *PagAS1a* regulates stem secondary growth. The results showed that secondary xylem development was significantly advanced in *PagAS1a* transgenic plants compared to WT (Fig. 3). Transcriptome analysis revealed that genes related to lignin and cellulose biosynthesis were significantly enriched in up-regulated DEGs in *PagAS1a* overexpression plants (Fig. 4), supporting the observed increase in secondary xylem development. Since fiber and vessel elements in the secondary xylem play essential roles in mechanical support and water conduction from roots to crown, the enhanced secondary xylem development may be the primary reason for the increased plant growth in *PagAS1a* transgenic plants. Notably, all functional analyses in this study were performed with *PagAS1a* overexpression and dominant repression transgenic plants, both of which were driven by the 35S promoter. The dominant repression of *PagAS1a* could cause functional interference of its interacting proteins. Therefore, the phenotype of *PagAS1a* transgenic plants may be partially due to *PagAS1a* ectopic expression and functional repression of PagAS1a interacting proteins. However, the significantly increased plant growth and secondary xylem development suggest that *PagAS1* genes are excellent candidate genes for breeding fast-growth trees with high wood production. In the future, it will be important to generate CRISPR/Cas9-mediated *PagAS1a*, *PagAS1b*, and *PagAS1c* single, double, or triple knockout mutants to further explore the functions of each *AS1* ortholog gene and their potential functional redundancy in *Populus*.

We also performed transcriptome analysis of gene expression changes caused by *PagAS1a* overexpression. Our results showed that the lignin biosynthetic process (GO:0009809) was significantly enriched in up-regulated DEGs while the lignin catabolic process (GO:0046274) was significantly enriched in down-regulated DEGs (Fig. 5a, b; Supplementary Tables S3, S4), which are consistent with the increased secondary xylem development and lignin content in *PagAS1a* overexpression plants (Fig. 3; Supplementary Fig. S4). We also carefully inspected the expression of genes related to cambium cell activity, and secondary phloem/xylem differentiation (Supplementary Fig. S5). Our results showed that the *PagLBD1, PtWOX4a, CLE41b,* and *XND1c* genes, which participate in regulating vascular cambium cell maintenance and secondary phloem/xylem differentiation, were significantly down-regulated in OE11 (Supplementary Fig. S3; Supplementary Table S6)^[59,60,62]^, while *PtrHB8* (promotes xylem development) and *XCP1* (specifically expressed in xylem vessel cells) were significantly up-regulated in OE11 (Supplementary Fig. S3; Supplementary Table S6)^[25,62,63]^. In Arabidopsis, AS1 is a key regulator in the establishment of leaf polarity, and AS1 forms a protein complex with the LOB domain transcription factor AS2 (AS1−AS2) and directly represses the expression of class I KNOX homeobox genes, which is critical for the establishment of leaf primordium^[29]^. In our transcriptome analysis, the class I KNOX homeobox genes, including *ARK1*, *ARK2*, and *KNAT2/6b*, which are important regulators of cambium cell maintenance and differentiation^[61,70,71]^, were down-regulated in *PagAS1a* overexpression plants (Supplementary Table S6). These results suggest that AS1 ortholog proteins may employ a similar regulatory module to promote cambium cell differentiation into xylem cells. In the future, it will be important to identify the direct target genes of PagAS1 through further experiments, as this will enhance our understanding of the molecular mechanisms underlying its function. Additionally, since AS1 functions in a protein complex with AS2 and other proteins in regulating leaf development, it will be informative to isolate PagAS1a-interacting proteins to reveal the molecular mechanisms underlying its function.

Phylogenetic analysis of *AS1* orthologs from 12 species revealed that they are divided into two distinct subgroups (Fig. 6). Subgroup I contains *AS1* orthologs from both monocot and dicot (including herbaceous and woody) species, while subgroup II exclusively contains orthologs from woody dicot species. This result indicates that subgroup I may have more conserved functions during evolution, whereas subgroup II expanded more recently and potentially gained new functions (Fig. 6). As there are no significant differences in *PagAS1a* transgenic plant leaves (Supplementary Fig. S2), it would be interesting to test whether *PagAS1c*, which belongs to subgroup I and has a closer distance to *Arabidopsis AS1*, functions in regulating leaf polarity establishment. In *Populus*, *PagAS1a,* and *PagAS1b* are two closely related orthologs belonging to subgroup II, suggesting that there might be functional redundancy between them. Notably, orthologs from monocots and dicots are divided into two subclusters within subgroup I, indicating that the *AS1* ancestral gene likely existed before the divergence of monocots and dicots (Fig. 6).

## Conclusions

In this study, we identified the *AS1* orthologs in *Populus*. Phylogenetic analysis indicated that the three *PagAS1* ortholog genes may have both common and divergent functions in *Populus*. Functional characterization of *PagAS1a* demonstrated that increasing its expression significantly promotes secondary xylem development and plant growth, providing a valuable candidate gene for breeding fast-growing trees for wood production. Notably, there were no significant morphological differences in leaves between *PagAS1a* transgenic plants and WT. It will be interesting to dissect whether *PagAS1b* or *PagAS1c* has a more prominent effect on leaf polarity and conduct functional comparison studies between *PagAS1a*, *PagAS1b*, and *PagAS1c* in the future.

## SUPPLEMENTARY DATA

Supplementary data to this article can be found online.

## Data Availability

The original RNA-sequencing data have been deposited into the Genome Sequence Archive at National Genomics Data Center, China National Center for Bioinformation/Beijing Institute of Genomics, Chinese Academy of Sciences (GSA: CRA024667) that are publicly accessible at https://ngdc.cncb.ac.cn/gsa.

## References

[1] (2010). Transcriptional regulation of secondary growth and wood formation. Journal of Integrative Plant Biology.

[2] (2010). Wood formation in angiosperms. Comptes Rendus Biologies.

[3] (2024). Woody plant cell walls: fundamentals and utilization. Molecular Plant.

[4] (2020). Architecture of a catalytically active homotrimeric plant cellulose synthase complex. Science.

[5] (2016). A single heterologously expressed plant cellulose synthase isoform is sufficient for cellulose microfibril formation *in vitro*. Proceedings of the National Academy of Sciences of the United States of America.

[6] (1999). Immunogold labeling of rosette terminal cellulose-synthesizing complexes in the vascular plant *Vigna angularis*. The Plant Cell.

[7] (2010). Characterization of cellulose synthase complexes in *Populus xylem* differentiation. New Phytologist.

[8] (2007). Genetic evidence for three unique components in primary cell-wall cellulose synthase complexes in *Arabidopsis*. Proceedings of the National Academy of Sciences of the United States of America.

[9] (2007). Organization of cellulose synthase complexes involved in primary cell wall synthesis in *Arabidopsis thaliana*. Proceedings of the National Academy of Sciences of the United States of America.

[10] (2018). Cellulose synthase stoichiometry in aspen differs from *Arabidopsis* and Norway spruce. Plant Physiology.

[11] (2020). Involvement of CesA4, CesA7-A/B and CesA8-A/B in secondary wall formation in *Populus trichocarpa* wood. Tree Physiology.

[12] (2010). Towards a systems approach for lignin biosynthesis in *Populus trichocarpa*: transcript abundance and specificity of the monolignol biosynthetic genes. Plant and Cell Physiology.

[13] (2002). Trends in lignin modification: a comprehensive analysis of the effects of genetic manipulations/mutations on lignification and vascular integrity. Phytochemistry.

[14] (2022). PtomtAPX is an autonomous lignification peroxidase during the earliest stage of secondary wall formation in *Populus tomentosa* Carr. Nature Plants.

[15] (2021). PtrLAC16 plays a key role in catalyzing lignin polymerization in the xylem cell wall of *Populus*. International Journal of Biological Macromolecules.

[16] (2023). Multiplex CRISPR editing of wood for sustainable fiber production. Science.

[17] (1999). Repression of lignin biosynthesis promotes cellulose accumulation and growth in transgenic trees. Nature Biotechnology.

[18] (2000). Essential role of caffeoyl coenzyme a O-methyltransferase in lignin biosynthesis in woody poplar plants. Plant Physiology.

[19] (2016). Knockdown of a laccase in *Populus deltoides* confers altered cell wall chemistry and increased sugar release. Plant Biotechnology Journal.

[20] (2020). LACCASE14 is required for the deposition of guaiacyl lignin and affects cell wall digestibility in poplar. Biotechnology for Biofuels.

[21] (2002). Laccase down-regulation causes alterations in phenolic metabolism and cell wall structure in poplar. Plant Physiology.

[22] (2014). Complexity of the transcriptional network controlling secondary wall biosynthesis. Plant Science.

[23] (2022). Lignin biosynthesis and accumulation in response to abiotic stresses in woody plants. Forestry Research.

[24] (2021). Functional identification of MdMYB5 involved in secondary cell wall formation in apple. Fruit Research.

[25] (2019). Phosphorylation of LTF1, an MYB transcription factor in *Populus*, acts as a sensory switch regulating lignin biosynthesis in wood cells. Molecular Plant.

[26] (2019). Hierarchical transcription factor and chromatin binding network for wood formation in *Populus trichocarpa*. The Plant Cell.

[27] (2015). PtoMYB92 is a Transcriptional activator of the lignin biosynthetic pathway during secondary cell wall formation in *Populus tomentosa*. Plant and Cell Physiology.

[28] (2019). The R2R3 MYB transcription factor MYB189 negatively regulates secondary cell wall biosynthesis in *Populus*. Tree Physiology.

[29] (2015). The complex of ASYMMETRIC LEAVES (AS) proteins plays a central role in antagonistic interactions of genes for leaf polarity specification in *Arabidopsis*. WIREs Developmental Biology.

[30] (2000). Asymmetric leaves1 mediates leaf patterning and stem cell function in *Arabidopsis*. Nature.

[31] (2003). Novel As1 and As2 defects in leaf adaxial-abaxial polarity reveal the requirement for ASYMMETRIC LEAVES1 and 2 and ERECTA functions in specifying leaf adaxial identity. Development.

[32] (2015). The ASYMMETRIC LEAVES complex employs multiple modes of regulation to affect adaxial-abaxial patterning and leaf complexity. The Plant Cell.

[33] (2005). The putative RNA-dependent RNA polymerase RDR6 acts synergistically with ASYMMETRIC LEAVES1 and 2 to repress BREVIPEDICELLUS and microRNA165/166 in *Arabidopsis* leaf development. The Plant Cell.

[34] (2001). The ASYMMETRIC LEAVES2 gene of *Arabidopsis thaliana* regulates formation of a symmetric *Lamina*, establishment of venation and repression of meristem-related homeobox genes in leaves. Development.

[35] (2025). ASYMMETRIC LEAVES1 promotes leaf hyponasty in Arabidopsis by light-mediated auxin signaling. Plant Physiology.

[36] (2013). MEGA6: molecular evolutionary genetics analysis version 6.0. Molecular Biology and Evolution.

[37] (2019). Genome-wide transcriptional adaptation to salt stress in *Populus*. BMC Plant Biology.

[38] (2014). A survey of *Populus* PIN-FORMED family genes reveals their diversified expression patterns. Journal of Experimental Botany.

[39] (2016). Transcript-level expression analysis of RNA-seq experiments with HISAT, StringTie and Ballgown. Nature Protocols.

[40] (2015). HTSeq—a Python framework to work with high-throughput sequencing data. Bioinformatics.

[41] (2010). edgeR: a Bioconductor package for differential expression analysis of digital gene expression data. Bioinformatics.

[42] (2021). MYB-mediated regulation of anthocyanin biosynthesis. International Journal of Molecular Sciences.

[43] (2001). The R2R3-MYB gene family in *Arabidopsis thaliana*. Current Opinion in Plant Biology.

[44] (2017). Identifying gene coexpression networks underlying the dynamic regulation of wood-forming tissues in *Populus* under diverse environmental conditions. New Phytologist.

[45] (2019). Evolution of coumaroyl conjugate 3-hydroxylases in land plants: lignin biosynthesis and defense. The Plant Journal.

[46] (2023). *OAF* is a *DAF*-like gene that controls ovule development in plants. Communications Biology.

[47] (2024). Influence of bagging on fruit quality, incidence of peel browning spots, and lignin content of 'Huangguan' pears. Plants.

[48] (2021). MYB transcription factors and its regulation in secondary cell wall formation and lignin biosynthesis during xylem development. International Journal of Molecular Sciences.

[49] (2015). NAC-MYB-based transcriptional regulation of secondary cell wall biosynthesis in land plants. Frontiers in Plant Science.

[50] (2021). Involvement of *Arabidopsis* multi-copper oxidase-encoding *LACCASE12* in root-to-shoot iron partitioning: a novel example of copper-iron crosstalk. Frontiers in Plant Science.

[51] (2023). Identification and function of miRNA-mRNA interaction pairs during lateral root development of hemi-parasitic *Santalum album* L. seedlings. Journal of Plant Physiology.

[52] (2024). Advances in plastic mycoremediation: focus on the isoenzymes of the lignin degradation complex. Science of The Total Environment.

[53] (2019). An AP2/ERF transcription factor ERF139 coordinates xylem cell expansion and secondary cell wall deposition. New Phytologist.

[54] (2020). Laccase3-based extracellular domain provides possible positional information for directing Casparian strip formation in *Arabidopsis*. Proceedings of the National Academy of Sciences of the United States of America.

[55] (2007). BLADE-ON-PETIOLE1 and 2 control *Arabidopsis* lateral organ fate through regulation of *LOB* domain and adaxial-abaxial polarity genes. The Plant Cell.

[56] (2011). Cell fate in the *Arabidopsis* root epidermis is determined by competition between WEREWOLF and CAPRICE. Plant Physiology.

[57] (2012). A FILAMENTOUS FLOWER orthologue plays a key role in leaf patterning in opium poppy. The Plant Journal.

[58] (2014). Regulation of cell fate determination by single-repeat R3 MYB transcription factors in *Arabidopsis*. Frontiers in Plant Science.

[59] (2010). Members of the LATERAL ORGAN BOUNDARIES DOMAIN transcription factor family are involved in the regulation of secondary growth in *Populus*. The Plant Cell.

[60] (2017). *WUSCHEL*-RELATED HOMEOBOX4 (*WOX4*)-like genes regulate cambial cell division activity and secondary growth in *Populus* trees. New Phytologist.

[61] (2019). *KNAT2/6b*, a class I KNOX gene, impedes xylem differentiation by regulating NAC domain transcription factors in poplar. New Phytologist.

[62] (2001). The *Arabidopsis* ATHB-8 HD-zip protein acts as a differentiation-promoting transcription factor of the vascular meristems. Plant Physiology.

[63] (2012). Genome-wide identification, evolutionary expansion, and expression profile of homeodomain-leucine zipper gene family in poplar (*Populus trichocarpa*). PLoS One.

[64] (2001). Role of PHABULOSA and PHAVOLUTA in determining radial patterning in shoots. Nature.

[65] (2011). The *Populus* Class III HD ZIP, popREVOLUTA, influences cambium initiation and patterning of woody stems. Plant Physiology.

[66] (2011). The *Populus* Class III HD ZIP transcription factor *POPCORONA* affects cell differentiation during secondary growth of woody stems. PLoS One.

[67] (2018). A HD-ZIP III gene, PtrHB4, is required for interfascicular cambium development in *Populus*. Plant Biotechnology Journal.

[68] (2013). PtrHB7, a class III HD-zip gene, plays a critical role in regulation of vascular cambium differentiation in *Populus*. Molecular Plant.

[69] (2017). AspWood: high-spatial-resolution transcriptome profiles reveal uncharacterized modularity of wood formation in *Populus tremula*. The Plant Cell.

[70] (2006). The *Populus homeobox* gene ARBORKNOX1 reveals overlapping mechanisms regulating the shoot apical meristem and the vascular cambium. Plant Molecular Biology.

[71] (2009). The *Populus homeobox* gene ARBORKNOX2 regulates cell differentiation during secondary growth. The Plant Journal.

