# Supplementary material for: The ASYMMETRIC LEAVES1 ortholog PagAS1a promotes xylem development and plant growth in Populus
Source: For Res (Fayettev). 2025 May 23;5:e010. doi: 10.48130/forres-0025-0011 (PMC12439027; doi:10.48130/forres-0025-0011)
Supplement: Supplementary file 1 — Supplementary data to this article can be found online. [file FR-2025-5-0011-Supplementary.zip › 10.48130_forres-0025-0011-Suppl-FigureS1.pdf]

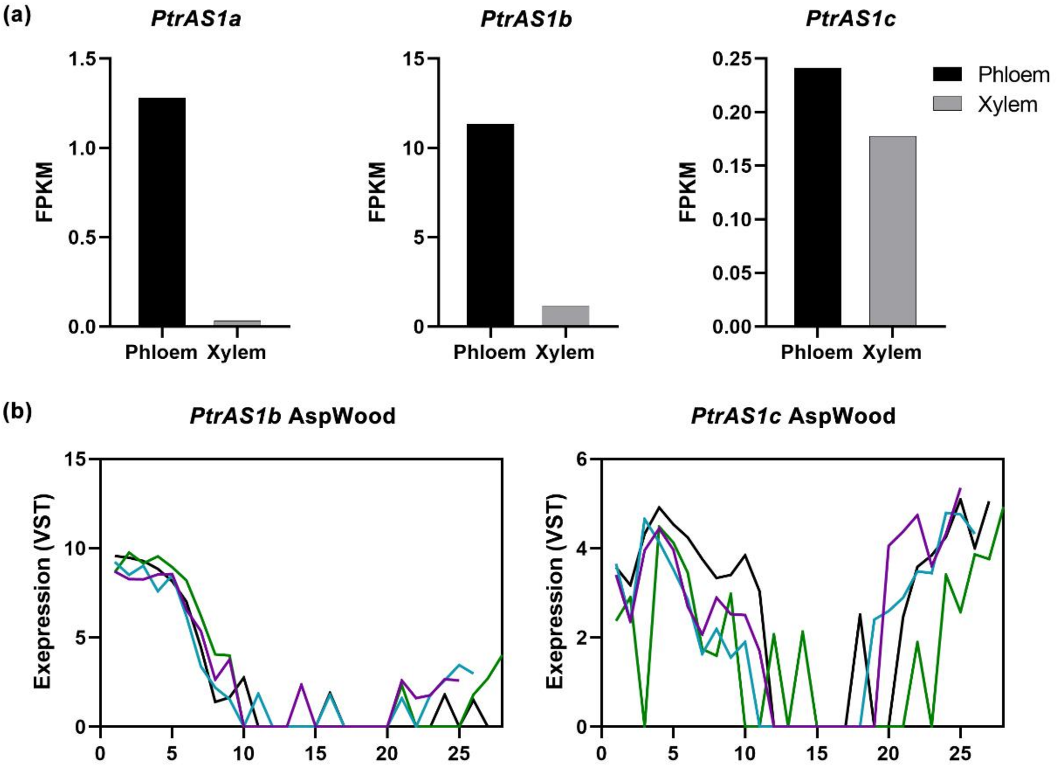

Fig. S1 Analysis of expression patterns of *PtrAS1a/b/c* genes. (a) Expression levels of *PtrAS1a/b/c* in secondary phloem and xylem from RNA-seq. (b) Expression patterns of *PtrAS1b/c* genes in vascular development. Four curves of different colors represent data from four wild aspen trees with the same genetic background. The average fragments per kilobase of exon per million fragments mapped (FPKM) for each gene from RNA-seq experiments are shown.
