# Supplementary material for: The ASYMMETRIC LEAVES1 ortholog PagAS1a promotes xylem development and plant growth in Populus
Source: For Res (Fayettev). 2025 May 23;5:e010. doi: 10.48130/forres-0025-0011 (PMC12439027; doi:10.48130/forres-0025-0011)
Supplement: Supplementary file 1 — Supplementary data to this article can be found online. [file FR-2025-5-0011-Supplementary.zip › 10.48130_forres-0025-0011-Suppl-FigureS2.pdf]

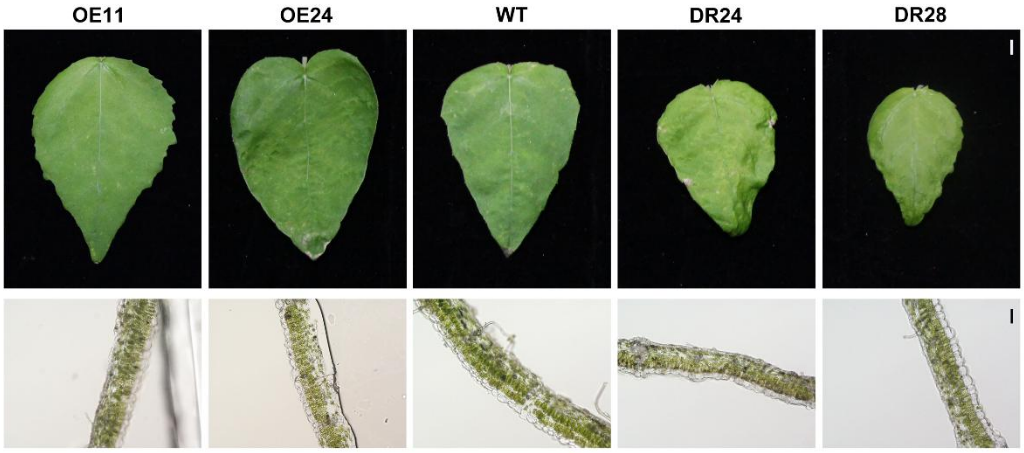

Fig. S2 Comparative analysis of leaves between WT and *PagAS1a* transgenic lines. Leaf and cross-sectional pictures of internode 6th of the plants. Scale bars: 0.5 cm for upper panels and 50 μm for lower panels.
