# Supplementary material for: The ASYMMETRIC LEAVES1 ortholog PagAS1a promotes xylem development and plant growth in Populus
Source: For Res (Fayettev). 2025 May 23;5:e010. doi: 10.48130/forres-0025-0011 (PMC12439027; doi:10.48130/forres-0025-0011)
Supplement: Supplementary file 1 — Supplementary data to this article can be found online. [file FR-2025-5-0011-Supplementary.zip › 10.48130_forres-0025-0011-Suppl-FigureS3.pdf]

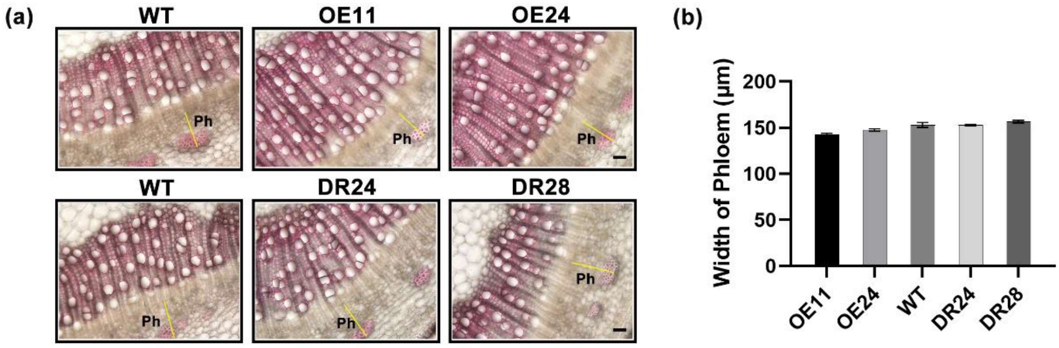


**Fig. S3** Phloem width identification of *PagAS1a* transgenic plants and WT. (a) Cross sectional images of the 13th stem internodes from WT, *PagAS1a*-OE and *PagAS1a*-SRDX plants stained with 0.1% phloroglucinol solution. (b) Phloem width of WT, *PagAS1a*-OE (OE11, OE24) and *PagAS1a*-SRDX (DR24, DR28) plants. Error bars represent SD. Scale bars: 50 μm.
