# Supplementary material for: The ASYMMETRIC LEAVES1 ortholog PagAS1a promotes xylem development and plant growth in Populus
Source: For Res (Fayettev). 2025 May 23;5:e010. doi: 10.48130/forres-0025-0011 (PMC12439027; doi:10.48130/forres-0025-0011)
Supplement: Supplementary file 1 — Supplementary data to this article can be found online. [file FR-2025-5-0011-Supplementary.zip › 10.48130_forres-0025-0011-Suppl-FigureS4.pdf]

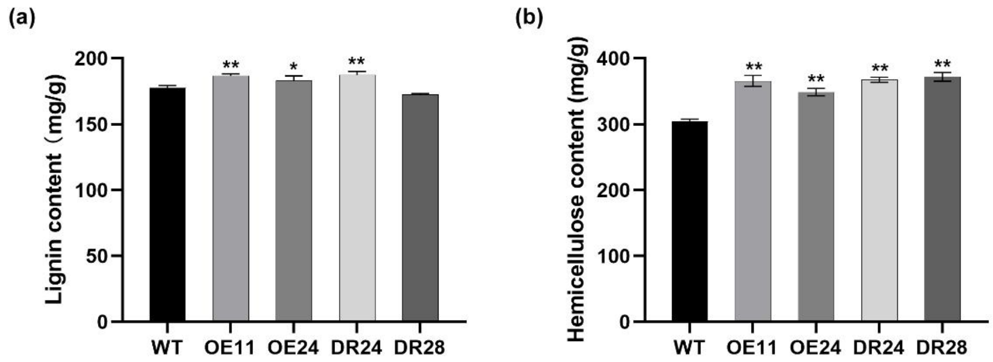

Fig. S4 Determination of lignin and hemicellulose contents. Lignin content (a), hemicellulose content (b) of *PagAS1a* transgenic plants and WT. Error bars represent SD. Asterisks indicate significant differences between WT and transgenic lines (Student's t-test): *$p < 0.05$, **$p < 0.01$.
