# Supplementary material for: The ASYMMETRIC LEAVES1 ortholog PagAS1a promotes xylem development and plant growth in Populus
Source: For Res (Fayettev). 2025 May 23;5:e010. doi: 10.48130/forres-0025-0011 (PMC12439027; doi:10.48130/forres-0025-0011)
Supplement: Supplementary file 1 — Supplementary data to this article can be found online. [file FR-2025-5-0011-Supplementary.zip › 10.48130_forres-0025-0011-Suppl-FigureS5.pdf]

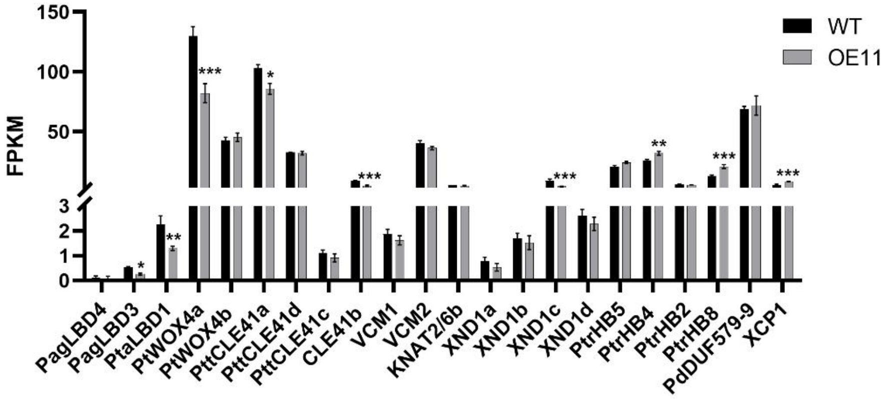

Fig. S5 Gene expression levels associated with cambium in WT and *PagAS1a* overexpression transgenic line (OE11) from RNA-seq. The average fragments per kilobase of exon per million fragments mapped (FPKM) for each gene from RNA-seq experiments are shown. Asterisks indicate significant differences between WT and *PagAS1a*-OE plants (Student's t-test): $*p < 0.05$; $**p < 0.01$; $***p < 0.001$.
